# MicroRNA-cytokine interplay and treg dysregulation in pediatric immune thrombocytopenia: implications for disease chronicity

**DOI:** 10.1007/s00431-026-07449-6

**Published:** 2026-10-21

**Authors:** Shaima S. Qassem, Doaa El Amrousy, Asmaa A. Ghalwash, Maha El Nady, Reem Awny, Amira Abdel-Kareem, Sarah Samy, Lamia M. Morad

**Affiliations:** 1 Clinical Pathology Department, Faculty of Medicine, Tanta University, Tanta, Egypt; 2 Pediatric department, Faculty of Medicine, Tanta University, Tanta, Egypt; 3 Medical Biochemistry Department, Faculty of Medicine, Tanta University, Tanta, Egypt; 4 Microbiology Department, Faculty of Medicine, Tanta University, Tanta, Egypt; 5 Public Health Department, Faculty of Medicine, Tanta University, Tanta, Egypt

**Keywords:** Pediatric ITP, MicroRNAs, Cytokines, Treg

## Abstract

This study aimed to investigate the immune-epigenetic profile of pediatric immune thrombocytopenic purpura (ITP) and its association with disease chronicity. This case–control study included 93 participants: 33 children with newly diagnosed ITP, 28 with chronic ITP (> 12 months), and 32 age- and sex-matched healthy controls. We assessed platelet count, serum transforming growth factor-β (TGF-β), interleukin-10 (IL-10) levels, circulating CD4⁺CD25⁺FoxP3⁺ Treg frequency, and relative expression of miR-106b-5p and miR-146a-5p. ITP patients demonstrated significantly lower platelet counts, Treg frequency, and TGF-β levels, with elevated IL-10 levels compared to controls. miR-106b-5p expression was significantly upregulated in ITP, particularly in chronic cases, and negatively correlated with platelet count, Treg frequency, and TGF-β. Conversely, miR-146a-5p was significantly downregulated and positively correlated with platelet count and Treg frequency. Chronic ITP exhibited more pronounced miRNA dysregulation and Treg depletion. ROC analysis revealed high diagnostic accuracy of both miRNAs for chronic ITP, with miR-146a-5p demonstrating superior sensitivity. *Conclusion:* This study highlights the interplay between immune dysregulation and epigenetics in ITP. The opposing roles of miR-106b-5p and miR-146a-5p in Treg function and cytokine homeostasis suggest their potential contribution to disease chronicity and their potential as chronicity biomarkers. These findings provide a foundation for future research aimed at improving ITP patient stratification, identifying therapeutic targets, and advancing precision-based interventions.

**Table:** 

**What is Known:** *• Despite extensive research, the aetiopathogenesis of immune thrombocytopenic purpura (ITP) remains incompletely understood, involving complex interactions between immune dysregulation, cytokine imbalance, and emerging epigenetic factors, such as microRNAs. * *• This work aimed to investigate the immunological and molecular mechanisms underlying ITP in the pediatric population, focusing on cytokine profiles (TGF-β, IL-10), regulatory T cells (Tregs), and the relative expression of miR-106b-5p and miR-146a-5p, and to assess their value as predictive tools for chronic ITP.*	
**What is New:** *• This study highlights the critical interplay between immune dysregulation and epigenetics in ITP. The opposing roles of miR-106b-5p and miR-146a-5p suggest their involvement in disease chronicity. These miRNAs may serve as non-invasive markers for ITP chronicity and as candidate targets for therapy to restore immune tolerance in ITP. Despite the cross-sectional nature of the current work, it provides a foundation for future research aimed at improving ITP patient stratification, identifying therapeutic targets, and advancing precision-based interventions. *

**What is Known:**

*• Despite extensive research, the aetiopathogenesis of immune thrombocytopenic purpura (ITP) remains incompletely understood, involving complex interactions between immune dysregulation, cytokine imbalance, and emerging epigenetic factors, such as microRNAs. *

*• This work aimed to investigate the immunological and molecular mechanisms underlying ITP in the pediatric population, focusing on cytokine profiles (TGF-β, IL-10), regulatory T cells (Tregs), and the relative expression of miR-106b-5p and miR-146a-5p, and to assess their value as predictive tools for chronic ITP.*

**What is New:**

*• This study highlights the critical interplay between immune dysregulation and epigenetics in ITP. The opposing roles of miR-106b-5p and miR-146a-5p suggest their involvement in disease chronicity. These miRNAs may serve as non-invasive markers for ITP chronicity and as candidate targets for therapy to restore immune tolerance in ITP. Despite the cross-sectional nature of the current work, it provides a foundation for future research aimed at improving ITP patient stratification, identifying therapeutic targets, and advancing precision-based interventions. *

## Introduction

Immune thrombocytopenia (ITP) is an autoimmune hematologic disorder defined by a peripheral platelet count below 100,000/μL affecting both pediatric and adult populations. ITP pathophysiology is multifactorial, including both humoral and cellular immune dysregulation. It involves immune-mediated destruction of circulating platelets as well as impaired thrombopoiesis due to autoantibody-induced cytotoxicity targeting megakaryocytes [1–3]. Moreover, there is a significant imbalance in T helper cell subsets, particularly a skewed Th1/Th2 cytokine profile. It is well established that Th1-dominant responses are characterized by elevated levels of pro-inflammatory cytokines, comprising interleukin-2 (IL-2), interferon-gamma (IFN-γ), and tumor necrosis factor-alpha (TNF-α), whereas Th2 responses are associated with the secretion of ILs 4, 5, 6,10, and 13 [3].

The ITP clinical course is stratified by duration into newly diagnosed (less than 3 months), persistent (from 3–12 months), and chronic (more than 12 months), with distinct prognostic implications and therapeutic approaches [4]. Children are typically presented with newly diagnosed ITP, whereas adults are more frequently diagnosed with chronic ITP [5]. Pediatric ITP is a self-limiting condition. Nonetheless, a subset of the affected pediatric population may experience severe mucosal bleeding necessitating hospitalization, drastically reducing health-related quality of life. Notably, approximately 20% of the affected pediatric population progresses to the chronic form [6, 7].

MicroRNAs (miRNAs) are small, non-coding RNAs about 19–25 nucleotides long, which are a cardinal part of the post-transcriptional regulatory machinery either by mRNA degradation or translational repression [8–10]. Dysregulation of miRNAs may disrupt the immune homeostasis by modulating the balance between T helper cell subsets, including (Th1/Th2) and (Th17/Treg) lineages. Furthermore, they may change Th9 and Th22 cell responses and activate T follicular helper (Tfh) cell-dependent auto-reactive B cells, thereby altering megakaryogenesis [11].

Concerning miRNA-106b-5p, microarray analysis revealed its upregulation in peripheral blood mononuclear cells (PBMCs) of ITP patients [12]. Previous records documented that this expression pattern is associated with intracellular transcription factor Nuclear Receptor Subfamily 4 Group A Member 3 (NR4A3) downregulation, a direct target involved in Treg differentiation through Forkhead Box P3 (Foxp3) signaling, thereby disrupting the balance between Treg and Th17 cells [13]. In addition, miR-146a-5p modulates the Treg/Th17 cell ratio through interleukin-1 receptor-associated kinase 1 (IRAK1) posttranscriptional inhibition, and its overexpression reduces Th17 and elevates Treg cells by suppressing IRAK1 [14].

Interleukin-10 (IL-10) is an immunoregulatory cytokine secreted by a specialized subset of CD4 T cells to mitigate inflammatory responses and maintain immune homeostasis [15–17]. Beyond IL-10, Tregs maintain immune tolerance through the release of anti-inflammatory cytokines, including transforming growth factor-β (TGF-β), and through direct cell–cell contact mechanisms. Additionally, it expresses Foxp3 that can be activated by both TGF-β and IL-2 [18].

Although dysregulation of these miRNAs has been described in ITP, their integrated relationship with Treg frequency and cytokine profiles in pediatric disease, particularly in relation to chronicity, remains insufficiently explored. Therefore, this study aimed to investigate the coordinated immunologic and epigenetic profile of pediatric ITP by evaluating Treg frequency, serum TGF-β and IL-10 levels, and the expression of miR-106b-5p and miR-146a-5p, with special emphasis on their association with disease chronicity.

### Patients and methods

This prospective case–control study was conducted at the Hematology Unit, Pediatric Department, Tanta University, in collaboration with the Medical Biochemistry, Microbiology, and Clinical Pathology Departments, Faculty of Medicine, Tanta University, Egypt, from June 2024 to June 2025. Ethical approval was granted by the Institutional Review Board of the Faculty of Medicine, Tanta University (permit code: 36264PR480/12/23). Written informed consent was obtained from the parents of enrolled children. Additionally, written assents for sharing were taken from all the parents of the included children. Procedures were strictly adherent to the Declaration of Helsinki II.

### Study population

A total of 93 participants were enrolled and divided into:

- newly diagnosed ITP group: 33 patients with newly diagnosed primary ITP (≤ 3 months),
- chronic ITP group: 28 patients with disease duration > 12 months,
- control group: 32 apparently healthy volunteers matched for age and sex with no history of hematologic or autoimmune disorders.

The diagnosis of ITP relied on the American Society of Hematology (ASH) criteria, which include: isolated thrombocytopenia with platelet counts below 100 × 10⁹/L, normal or increased megakaryocytes in bone marrow aspirate (if performed), and exclusion of secondary causes such as autoimmune disease, viral infections, or drug-induced thrombocytopenia.

Inclusion Criteria: Pediatric patients aged below 18 years with a confirmed diagnosis of acute or chronic primary ITP. No corticosteroid, thrombopoietin receptor agonist, or immunosuppressive treatment for at least 3 months before enrollment.

Exclusion Criteria: Children with secondary ITP attributable to systemic autoimmune disease, infections (HIV, HBV, HCV, CMV), malignancy, immune deficiency (immunoglobulin and cellular markers), or recent blood transfusion (within 1 month) were excluded.

### Blood sampling and laboratory investigations

Venous blood samples were collected using strict aseptic technique from all participants. Samples were divided into two groups; the first was placed into ethylene diamine tetra-acetic acid (EDTA)-treated tubes for hematological analyses, flow cytometry, and molecular assays. The second was transferred into sterile plain centrifuge tubes, allowed to clot at room temperature, and then immediately centrifuged at 1000 × g for 15 min. Sera were separated, aliquoted, and stored at −20 °C until biochemical studies.

1. Complete blood count was immediately measured using an automated hematology analyzer (ERMA INC, japan).
2. Cytokines assays: serum TGF-β and IL-10 levels were assessed using an enzyme-linked immunosorbent assay (ELISA) kit provided by Abcam, UK., Catalog no. ab100647 and ab185986, respectively, following the manufacturer’s protocol.
3. Microrna analysis:
  3.1 RNA extraction: Total RNA, including small RNAs, was isolated using the QIAGEN miRNeasy kit following the protocol provided. Isolated RNA concentration and quality were evaluated by monitoring OD 260 and the OD 260/280 ratio, respectively, using a NanoDrop.
  3.2 Reverse transcription: Complementary DNA (cDNA) synthesis was carried out using a Reverse Transcription QIAGEN Kit specific for microRNAs.
  3.3 Quantitative real-time polymerase chain reaction (qRT-PCR): Expression of miR-106b-5p and miR-146a-5p was quantified by RT-PCR (Rotor-Gene Q, QIAGEN) using SYBR Green chemistry. U6 small nuclear RNA was used as an endogenous control. Sequence-specific primers were synthesized according to published sequences. For miR-106b-5p, the forward primer sequence was 5′-TAAAGTGCTGACAGTGCAGAT-3′. For miR-146a-5p, the forward primer sequence was 5′- TGAGAACTGAATTCCATGGGTT-3′. In both cases, a reverse primer with the sequence 5′-GTGCAGGGTCCGAGGT-3′ was employed. For U6 small nuclear RNA, the forward primer was 5′-CTCGCTTCGGCAGCACA-3′, while the reverse primer was 5′- AACGCTTCACGAATTTGCGT-3′. All were optimized for use with SYBR Green-based qRT-PCR.
    The thermal cycling was conducted following this protocol: an initial denaturation step at 95 °C for 10 min, followed by 40 to 45 cycles consisting of DNA denaturation at 95 °C for 15 s, primer annealing at 60 °C for 30 s, and DNA extension at 72 °C for 30 s. A melting curve was generated and analyzed by gradually increasing the temperature to 95 °C at the end of the final cycle. Cycle threshold (Ct) values were determined for the target genes and U6, and relative expression levels were calculated using the 2-ΔΔCT method [19].
4. Flow cytometry for T regulatory (Treg) cells: Flow cytometry was used to measure the percentage of CD4 + CD25 + FoxP3 + Treg cells in the peripheral blood of participants. Cells were stained with fluorochrome-conjugated monoclonal antibodies against CD4 (FITC), CD25 (PE), and FoxP3 (APC) to identify Treg lymphocytes. Acquisition was performed on a BD FACS-Canto II cytometer with FACSDiva 9.0^5^. Monoclonal antibodies provided by BD Biosciences, US.

Tregs were defined as CD4⁺CD25⁺FoxP3⁺ lymphocytes and expressed as a percentage of total CD4⁺ T cells.

### Statistical analysis

Results were analyzed using the Statistical Package for the Social Sciences SPSS version XX, IBM Corp., Armonk, NY, USA). The results of the current work were presented as means and standard deviations (SD) if the data were quantitative, and as numbers and percentages if the data were categorical. Intergroup comparisons were performed using one-way ANOVA or Kruskal–Wallis test with post hoc analysis. Correlations between microRNA expression, platelet count, cytokine levels, and Treg percentages were assessed using Spearman’s rank correlation coefficient. The receiver operating characteristics (ROC) curve was drawn to assess the predictive value of miR-106b-5p and miR-146a–5p to diagnose patients with chronic ITP. A p-value < 0.05 was considered statistically significant.

## Results

We screened 100 patients with ITP; 39 patients were excluded, and 61 children met the inclusion criteria and were divided into newly diagnosed ITP and chronic ITP. There was no significant difference as regards age and sex among the study groups (Patients and healthy control). However, it is noteworthy that each of the newly diagnosed and chronic ITP cases was more common in females than in males. Also, there was significant difference as regards age between newly diagnosed ITP and chronic ITP patients.

Complete blood count (CBC) demonstrated that platelet counts were significantly lower in the patient groups, especially those with newly diagnosed ITP, while the mean platelet volume (MPV) was significantly higher among ITP patients compared to the healthy controls. Hemoglobin, hematocrite, and total leukocytic count (TLC) remained comparable across groups. (Table 1).

**Table 1:** Demographic and laboratory data in the studied groups

Variables	Group I	Group II	Group III	P
Age (years)	9.1 ± 4.2	11.5 ± 3.6	9.8 ± 4.5	0.085
Sex (male: female)	(12: 21)	(10: 18)	(12: 20)	0.989
Hemoglobin (g/dl)	11.8 ± 0.6	11.7 ± 0.5	12.0 ± 0.3	0.257
Hematocrit	35.4 ± 2.3*	34.1 ± 2.6*	34.1 ± 2.4	0.113
MCH	30.6 ± 2.3	30.0 ± 23.0	31.2 ± 2.5	0.184
MCV	78.3 ± 3.5	78.4 ± 2.6	79.9 ± 6.1	0.272
MPV	12.1 ± 1.3	11.0 ± 1.0	8.7 ± 0.5	0.001
TLC	7621.2 ± 1703.4	7464.3 ± 1574.6	8281.3 ± 1694.1	0.128
Platelets	43.2 ± 12.6	80.1 ± 15.9	228.8 ± 60.9	0.001
TGF-β (pg/ml)	2271.5 ± 269.5	1065.7 ± 132.4	3290.1 ± 401.0	0.001
IL- 10 (pg/ml)	15.2 ± 3.0	10.6 ± 2.2	6.3 ± 1.1	0.001
miR-106b-5p	2.1 ± 0.4	2.7 ± 0.6	0.9 ± 0.4	0.001
miR-146a–5p, relative expression level	3.6 ± 1.0	2.8 ± 0.8	7.4 ± 1.4	0.001
Treg, % (from PB CD4 + T cells)	4.7 ± 0.9	3.4 ± 0.7	8.7 ± 1.5	0.001

MCH: mean corpuscular hemoglobin, MCV: mean corpuscular volume, MPV: mean platelet volume, TLC: total leucocytic count, TGF-β: transforming growth factor-β, IL-10: interleukin-10

Patients with chronic ITP exhibited statistically significantly lower levels of serum TGF-β compared to newly diagnosed ITP patients. In contrast, serum IL-10 levels were significantly elevated in both newly diagnosed and chronic ITP patients compared to the healthy controls. Moreover, miR-106b-5p expression was significantly upregulated in newly diagnosed and chronic ITP patients compared to the control group (*P* = 0.001). Interestingly, chronic ITP patients exhibited a pronounced decrease in miR-146a-5p levels compared to those with newly diagnosed ITP. Moreover, patients with chronic ITP showed a profound decrease in Treg frequency in comparison to their newly diagnosed counterparts **(**Table 1).

Cutaneous bleeding was the most common clinical presentation in newly diagnosed ITP patients (75.8%) followed by mucosal bleeding (24.2%). The majority of chronic ITP patients had no bleeding (67.9%). As regards therapy of newly diagnosed ITP patients, 24.4% were managed conservatively, and 57.6% received steroids. In patients with chronic ITP, 10.7% received steroids, 53.6% received combined steroids and thrombopoietin receptor agonist (TPOS), and 35.7% received combined steroids, TPOS, and immunosuppressants (Table 2).

**Table 2:** Clinical bleeding history, Buchanan score, and treatment history among studied patients

	Group I		Group II		χ^2^	P
	N	%	N	%		
**Bleeding severity**						
**No bleeding**	0	0.0%	19	67.9%	32.534	0.001
**Cutaneous bleeding**	25	75.8%	7	25.0%		
**Mucosal bleeding**	8	24.2%	2	7.1%		
**Buchanan score**						
**Zero**	0	0.0%	19	67.9%	32.534	0.001
**1–2**	25	75.8%	7	25.0%		
**3**	8	24.2%	2	7.1%		
**Treatment**						
**Observation**	19	57.6%	0	0.0%		
**Steroid**	14	42.4%	3	10.7%	51.051	0.001
**Steroid and TPOS**	0	0.0%	15	53.6%		
**Steroid, TPOS and Immunosuppressants**	0	0.0%	10	35.7%		

χ2: Chi-Square test, TPOS: thrombopoietin receptor agonist

Correlation analyses were performed, and miR-106b-5p demonstrated a significant negative correlation with platelet count, serum TGF-β levels, and Treg %, while exhibiting a significant positive correlation with IL-10 serum levels. On the other hand, miR-146a-5p was positively correlated with platelet count, TGF-β, and Treg%, but inversely correlated with IL-10 (Table 3).

**Table 3:** Correlation of miR-106b-5p and miR-146a-5p with different variables

Variables	miR-106b-5p		miR-146a–5p	
	r	P	r	P
**Platelet count**	−0.652	0.001	0.741	0.001
**TGF-β (pg/ml)**	−0.797	0.001	0.744	0.001
**IL- 10 (pg/ml)**	0.54	0.001	−0.58	0.001
**Treg, %**	−0.765	0.001	0.770	0.001

TGF-β: transforming growth factor-β, IL-10: interleukin-10

We performed additional age-stratified and age-adjusted analyses. Chronic ITP patients were significantly older than newly diagnosed patients: mean age was 11.5 ± 3.6 versus 9.1 ± 4.2 years (*p* = 0.023), and 71.4% of chronic patients versus 39.4% of newly diagnosed patients were aged ≥ 10 years (*p* = 0.012).

Patients were subsequently stratified into < 10 years and ≥ 10 years. In children < 10 years, chronic ITP remained associated with higher platelet counts (85.0 vs 43.0 × 10⁹/L, *p* < 0.001), lower TGF-β (1023 vs 2390 pg/mL, *p* < 0.001), lower IL-10 (1.3 vs 4.8 pg/mL, *p* < 0.001), and lower Treg percentage (3.5% vs 4.7%, *p* = 0.002). Similar differences were observed among patients aged ≥ 10 years: platelet count 78.5 vs 49.0 × 10⁹/L, TGF-β 1080 vs 2000 pg/mL, IL-10 1.4 vs 4.5 pg/mL, and Tregs 3.2% vs 4.8%; all *p* < 0.001.

Furthermore, after adjustment for continuous age, chronicity remained significantly associated with platelet count, TGF-β, IL-10, both microRNA measures, and Treg percentage. Importantly, none of the age-group × disease-course interaction terms were significant, indicating that the principal immunological differences were not explained by age. These analyses were provided as Tables (4, 5, 6 and 7).

**Table 4:** Age profile of newly diagnosed and chronic ITP patients

Age measure	Newly diagnosed ITP (*n* = 33)	Chronic ITP (*n* = 28)	P
**Age at blood sampling**	9.1 ± 4.2	11.5 ± 3.6	0.023
**Age categories**			
Age < 10 years, n (%)	20 (60.6)	8 (28.6)	0.012
Age ≥ 10 years, n (%)	13 (39.4)	20 (71.4)	

ITP: immune thrombocytopenia

**Table 5:** Laboratory findings in patients aged < 10 years, stratified by ITP course

Outcome	Newly diagnosed ITP Median (IQR) *n* = 20	Chronic ITP median (IQR) *n* = 8	P
Platelet count (× 10⁹/L)	43.0 (31.0–50.0)	85.0 (70.2–88.5)	< 0.001
TGF-β (pg/mL)	2390.0 (2190.0–2570.0)	1023.0 (1004.5–1092.5)	< 0.001
IL-10 (pg/mL)	4.8 (4.0–6.2)	1.3 (1.0–2.0)	< 0.001
miR-106b-5p (relative expression)	2.0 (1.9–2.4)	2.3 (2.1–2.7)	0.131
miR-155–5p (relative expression)	3.8 (2.9–4.2)	2.6 (2.0–3.0)	0.023
Treg (% of PB CD4 + T cells)	4.7 (3.9–5.1)	3.5 (2.9–4.0)	0.002

ITP: immune thrombocytopenia, IQR: interquartile range, TGF-β: transforming growth factor-β, IL-10: interleukin-10, IQR: interquartile range, PB: peripheral blood

**Table 6:** Laboratory findings in patients aged ≥ 10 years, stratified by ITP course

Outcome	Newly diagnosed ITP median (IQR) *n* = 13	Chronic ITP median (IQR) *n* = 20	P
Platelet count (× 10⁹/L)	49.0 (38.0–50.0)	78.5 (68.0–90.0)	< 0.001*
TGF-β (pg/mL)	2000.0 (2000.0–2400.0)	1080.0 (949.0–1190.0)	< 0.001*
IL-10 (pg/mL)	4.5 (4.4–5.3)	1.4 (1.1–2.0)	< 0.001*
miR-106b-5p (relative expression)	2.3 (1.7–2.4)	2.8 (2.5–3.3)	0.002*
miR-155–5p (relative expression)	3.5 (2.8–4.1)	3.0 (2.1–3.5)	0.086
Treg (% of PB CD4 + T cells)	4.8 (4.1–6.0)	3.2 (2.8–3.9)	< 0.001*

ITP: immune thrombocytopenia, IQR: interquartile range, TGF-β: transforming growth factor-β, IL-10: interleukin-10

**Table 7:** Associations between chronic ITP and laboratory measures adjusted for age at blood sampling

Outcome	Adjusted β	95% CI	P for chronicity	P for age	P for interaction
Platelet count (× 10⁹/L)	35.99	28.38 to 43.60	< 0.001	0.392	0.468
TGF-β (pg/mL)	−1172.35	−1287.77 to −1056.93	< 0.001	0.047	0.060
IL-10 (pg/mL)	−3.50	−4.01 to −3.00	< 0.001	0.804	0.784
miR-106b-5p (relative expression)	0.60	0.30 to 0.90	< 0.001	0.785	0.183
miR-155–5p (relative expression)	−0.84	−1.34 to −0.35	0.001	0.266	0.513
Treg (% of PB CD4 + T cells)	−1.39	−1.86 to −0.92	< 0.001	0.446	0.495

Each row represents a separate linear regression model comparing chronic with newly diagnosed ITP and adjusting for continuous age at sampling. Positive β values indicate higher values in chronic ITP. HC3 heteroscedasticity-consistent standard errors were used. Interaction P values are from separate models containing disease course, age stratum (< 10 versus ≥ 10 years), and their product term. CI, confidence interval, TGF-β: transforming growth factor-β, IL-10: interleukin-10

**Fig. 1:**
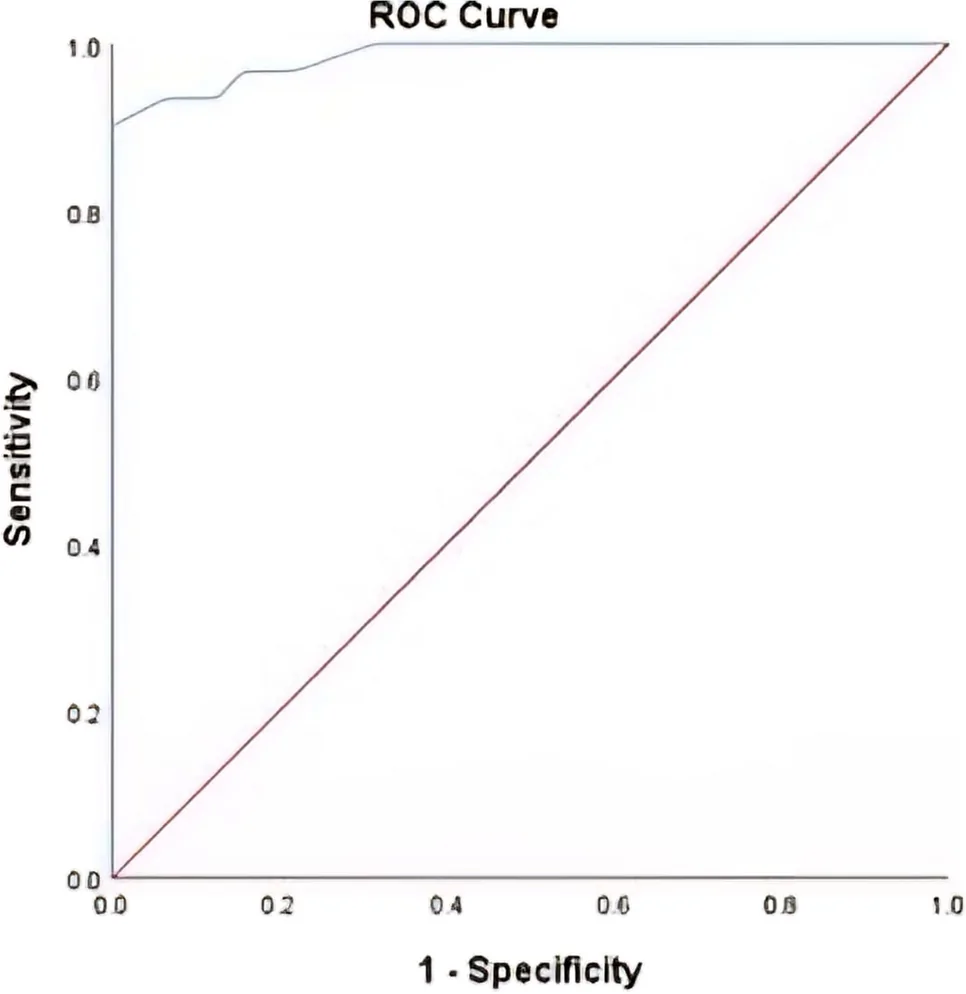
ROC curve of miR-106b-5p to predict patients with chronic ITP

**Fig. 2:**
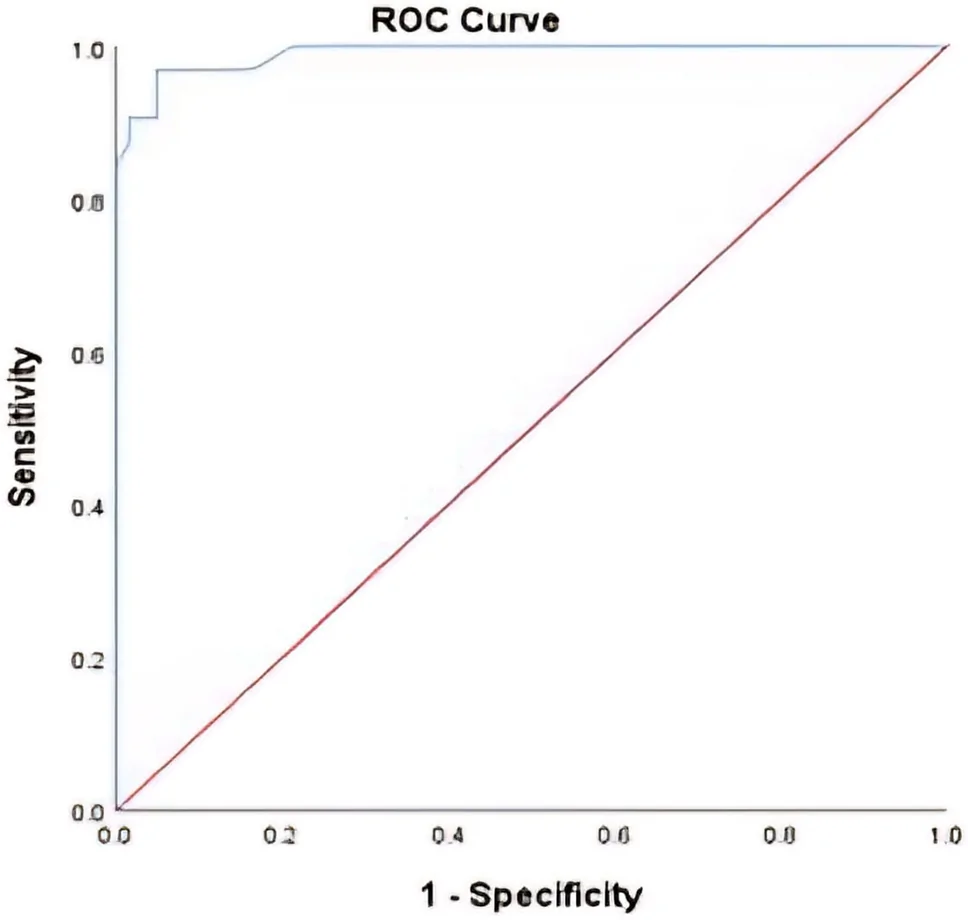
ROC curve of miR-146a–5p to predict patients with chronic ITP

The ROC curve analysis revealed that miR-106b-5p and miR-146a-5p have cutoff values of 0.986 and 0.991, with sensitivities of 93.4% and 96.9%, and specificities of 93.7% and 95.1%, respectively, to predict patients with chronic ITP. (Figs. 1 and 2).

## Discussion

ITP is an autoimmune disease with isolated thrombocytopenia. Despite being the subject of extensive research, its exact aetiopathogenesis remains not fully elucidated. It is hypothesized to involve numerous environmental factors, including viral infection and immunologic triggers. Away from triggers, ITP is primarily driven by a breakdown in self-tolerance, involving both cellular and humoral immune dysregulation, which results in the immune system targeting platelets [20].

In the present study, platelet count analysis revealed that the newly diagnosed ITP patients had markedly lower counts than chronic cases. This should be interpreted in light of evidence indicating that a higher platelet count at diagnosis is a predictive risk factor for chronicity in pediatric ITP, as supported by previous studies [21–23]. The hallmark feature of ITP is thrombocytopenia, which can be attributed to multiple pathomechanics, including anti-platelet autoantibody-mediated destruction by monocytes and macrophages of the reticuloendothelial system, increased platelet apoptosis, and impaired megakaryopoiesis [24, 25]. Conversely, in line with El Zaiat et al. [10], MPV was markedly elevated in ITP patients compared with controls, with higher values in the newly diagnosed cases than their chronic counterparts. This reflects compensatory bone marrow stimulation in response to the peripheral platelet destruction, producing younger, larger platelets, a phenomenon supported by other investigators [26, 27].

The existing literature highlights antiplatelet autoantibodies, dysregulated immune responses, and an altered cytokine milieu as hidden drivers of the pathogenesis and chronicity of ITP. Dysregulation of the Treg/Th17 balance contributes to sustained immune activation and platelet destruction. TGF-β and IL-10, key immunoregulatory cytokines involved in maintaining peripheral tolerance, have been reported to be dysregulated in ITP [28].

Regarding the cytokine profile, the current work revealed significantly reduced serum TGF-β levels in ITP patients compared to healthy controls, with higher concentrations recorded in the newly diagnosed cases relative to their chronic counterparts. Consistent with our findings, former workers [29–31] documented diminished TGF-β levels in ITP, which increased following therapeutic intervention. These observations suggest a potential association between TGF-β restoration and disease remission. In contrast, our data significantly demonstrated elevated serum IL-10 levels in ITP patients compared with controls, with higher concentrations in newly diagnosed than in chronic ones. This observation aligns with the findings of Goelz et al. [32], who demonstrated increased pro-inflammatory cytokines, including tumor necrosis factor-alpha (TNF-α) and IL-15, and anti-inflammatory cytokines, including interleukin-1 receptor antagonist (IL-1Ra), IL-10, and interferon gamma-induced protein 10 (IP-10) in newly diagnosed ITP. In contrast, Soliman et al. [33] recorded no significant overall difference in IL-10 levels between ITP patients and controls, attributing this discrepancy to IL-10 promoter polymorphisms (1082 AA, 592 AA genotypes) and/or differences in cohort composition. Taken together, these findings suggest that cytokine profiles in ITP may vary according to the disease stage and the patient's genetic background, contributing to interstudy heterogeneity. In parallel, we observed significantly reduced circulating Treg cells in patients with ITP compared to the control group, with greater depletion and more functional impairment in chronic ITP patients. This supports the findings of other investigators [10, 34], who linked Treg deficiency and impaired cytokine production (IL-10, TGF-β) to disease chronicity and treatment resistance.

At the molecular level, microRNAs act as key post-transcriptional regulators of immune cell development and function. Notably, miR-146a-5p and miR-106b-5p have emerged as potential epigenetic regulators of Treg/Th17 homeostasis. So, investigating the interplay between these epigenetic modulators and ITP-associated immune dysregulation may provide deeper insights into the mechanisms driving ITP progression and chronicity [13, 35].

Our study demonstrated significant upregulation of miR-106b-5p in ITP patients compared with the control group, with markedly higher expression in chronic cases. Our findings are in harmony with previous reports. Qian et al. [36] observed significant upregulation of miR-106b-5p and miR-200c-3p in ITP, particularly in the chronic form. Similarly, El Zaiat et al. [10] reported elevated plasma levels of both miRNAs in ITP groups relative to controls. Li et al. [13] further confirmed miR-106b-5p upregulation in pediatric ITP and demonstrated that it targets NR4A3, resulting in FOXP3 suppression, impaired regulatory T cell (Treg) differentiation, and disruption of the Treg/Th17 balance central to ITP pathogenesis. Collectively, these findings highlight miR-106b-5p as a potential driver of chronic disease and a candidate biomarker for ITP chronicity. Conversely, our data revealed significant downregulation of miR-146a-5p in ITP patients compared with the control group, with notable differences across the acute and chronic subgroups. This aligns with the results of other researchers [10, 37], who reported decreased miR-146a-5p expression in both newly diagnosed and chronic ITP patients. Given the established role of miR-146a-5p in dampening excessive immune activation, its reduced expression may contribute to the persistent immune dysregulation observed in ITP.

Collectively, our findings highlight the opposing immunomodulatory roles of miR-106b-5p and miR-146a-5p in ITP pathogenesis, influencing Treg function, cytokine balance, and platelet production. Their dysregulation appears closely tied to disease chronicity and severity, supporting their potential as biomarkers of progression and therapeutic targets for restoring Treg-mediated immune tolerance. The correlation analyses further support these mechanistic links. Wang et al. [38] reported upregulated miR-106b-5p driving Th17/Treg in children with ITP, our data extend this by revealing a significant negative correlation between platelet count and miR-106b-5p. We also found negative correlations between miR-106b-5p and both Treg frequency and TGF-β levels, corroborating the results of Xie et al. [39] and Li et al. [13], who showed that miR-106b-5p targets FOXP3 and NR4A3, thereby suppressing Treg differentiation and TGF-β production, and driving the Treg/Th17 imbalance in ITP. Interestingly, a positive correlation with IL-10 was also observed, possibly reflecting a compensatory anti-inflammatory response in the setting of heightened immune dysregulation, though this warrants further investigation. Moreover, miR-146a-5p relative expression was positively correlated with platelet count, TGF-β levels, and Treg frequency, and negatively correlated with IL-10. These findings are consistent with Liu et al. [35]. This suggests that reduced miR-146a-5p may contribute to the ITP-associated impaired Treg homeostasis and thrombocytopenia, while its restoration could potentially support immune tolerance and platelet recovery.

Taken together, our findings reveal a complex interplay between platelet indices, cytokine milieu (IL-10, TGF-β), Treg deficiency, and dysregulated miRNA expression (miR-106b-5p, miR-146a-5p) in ITP pathogenesis. This integrated profile appears closely linked to disease chronicity and severity, highlighting these factors as potential biomarkers of disease progression and therapeutic targets aimed at restoring Treg-mediated immune tolerance.

ROC analysis revealed that both miR-106b-5p and miR-146a-5p had good predictive value for diagnosing chronic ITP with sensitivities of 93.4% and 96.9%, and specificities of 93.7% and 95.1%, respectively. Notably, miR-146a-5p demonstrated slightly higher sensitivity, positioning it as a particularly promising marker for early detection of cases with chronic ITP.

These findings agree with Qian et al. [36], who identified miR-106b-5p as a potential prognostic biomarker for chronic ITP, with its overexpression differentiating patients with chronic ITP. Similarly, El Zaiat et al. [10] found that miR-106b-5p, with a cutoff value ≤ 6.163, could significantly predict complete treatment response with modest sensitivity and specificity (AUC = 0.893), while miR-146a-5p showed no predictive value for treatment response in these patients (AUC = 0.595).

It is worth noting, though miR-146a-5p is recognized as a potential therapeutic target and mechanistic biomarker in ITP, current literature lacks data on its diagnostic sensitivity or specificity in chronic ITP. The existing reports focus on its functional effects and mechanistic pathways rather than on its diagnostic performance. However, reports in other diseases, such as epilepsy and amyotrophic lateral sclerosis, have evaluated miR-146a-5p as a diagnostic marker with moderate diagnostic accuracy (AUC = 0.71 in epilepsy) [40].

Intriguingly, despite the cross-sectional nature, the distinct cytokine profiles, Treg dysregulation, and disease-associated microRNA signatures identified herein may represent biologically informative molecular signatures with significant translational potential. The combined assessment of miRNA expression profiles and immune parameters may contribute to the stratification of patients with ITP and facilitate the identification of individuals exhibiting a more persistent immune-dysregulated disease phenotype. Furthermore, serial monitoring of these biomarkers may provide valuable insights into dynamic changes in immune status during treatment and potentially help patients monitoring via identification of treatment-associated immunological patterns. Finally, the molecular pathways involving miR-106b-5p and miR-146a-5p may represent promising areas for future mechanistic investigation and could potentially provide novel insights into the pathophysiology of ITP.

### Limitations of the study

While the current work offers important insights, several limitations must be acknowledged. First, the small number of participants could constrain the strength and external validity of the study outcomes. The relatively small sample size may have limited the statistical power to detect certain associations. For instance, although the 3 month-treatment free criterion reduces the influence of recent therapies, prior treatment exposure may still have affected the immunological and epigenetic profiles evaluated in the study. So, larger future studies with larger population are warranted to allow exploring the impact of previous therapeutic approaches and consider performing a subgroup analysis stratified by treatment history. Also, prospective longitudinal studies in treatment-naïve patients are recommended to distinguish disease-related from treatment-related immunological changes. Second, the cross-sectional nature of the study restricts our ability to infer causality or assess the long-term prognostic significance of microRNA and cytokine profiles in ITP. Longitudinal research is essential to unravel their dynamic roles in progress monitoring and chronicity, as well as their potential as early indicators of chronicity. Third, being a single-center design may constrain the diversity of the study population. Future studies should consider a more comprehensive panel of microRNAs and cytokines, and ideally adopt a prospective design to explore treatment responses over time. Addressing these limitations will be critical for advancing our understanding of the signature of this crosstalk in ITP chronicity and progress monitoring.

## Conclusion

This study identifies a coordinated immuno-epigenetic profile in pediatric ITP characterized by regulatory T-cell depletion, reduced TGF-β levels, elevated IL-10, and opposing dysregulation of miR-106b-5p and miR-146a-5p. The more pronounced alterations detected in chronic ITP suggest that miR-106b-5p upregulation and miR-146a-5p downregulation may reflect mechanisms contributing to disease persistence rather than representing isolated molecular changes. These findings underscore the potential relevance of these miRNAs as biomarkers of immune dysregulation and chronicity. Importantly, despite its cross-sectional nature, this study provides a valuable foundation for future research, which could provide a mechanistic framework for refining patient stratification, identifying novel therapeutic targets, and development of precision-based interventions tailored to specific immunological and molecular phenotypes.

## Data Availability

all the data of the study are available from the corresponding author on reasonable request.

## Abbreviations

ASH: American Society of Hematology
AUC: Area under the curve
CBC: Complete blood count
EDTA: Ethylene diamine tetra-acetic acid
ELISA: Enzyme-linked immunosorbent assay
IL: Interleukin
IL-1Ra: Interleukin-1 receptor antagonist.
IRAK1: Interleukin-1 receptor-associated kinase 1
ITP: Immune thrombocytopenia.
MiRNAs: MicroRNAs
MPV: Platelet volume
PBMCs: Peripheral blood mononuclear cells
qRT-PCR: Quantitative real-time polymerase chain reaction
ROC: Receiver operating characteristics
SD: Standard deviation
TGF-β: Transforming growth factor-β
TNF-α: Tumor necrosis factor-alpha.
TLC: Total leukocytic count

## References

[1] Mititelu A, Onisâi MC, Roșca A, Vlădăreanu AM (2024) Current understanding of immune thrombocytopenia: a review of pathogenesis and treatment options. Int J Mol Sci 25(4):2163 10.3390/ijms25042163 PMC10889445 38396839

[2] LeVine DN, Brooks MB (2019) Immune thrombocytopenia (ITP): pathophysiology update and diagnostic dilemmas. Vet Clin Pathol 48(Suppl 1):17–28 10.1111/vcp.12774 31538353

[3] Andreescu M (2023) The link between immune thrombocytopenia and the cytokine profile: a bridge to new therapeutical targets. Front Hematol 2:1191178

[4] Efficace F, Mandelli F, Fazi P, Santoro C, Gaidano G, Cottone F, Mazzucconi MG (2016) Health-related quality of life and burden of fatigue in patients with primary immune thrombocytopenia by phase of disease. Am J Hematol 91(10):995–1001 10.1002/ajh.24463 27351715

[5] Nomura S (2016) Advances in diagnosis and treatments for immune thrombocytopenia. Clin Med Insights Blood Disord 9:CMBD.S39643 10.4137/CMBD.S39643 PMC4948655 27441004

[6] Grainger JD, Kühne T, Hippenmeyer J, Cooper N (2021) Romiplostim in children with newly diagnosed or persistent primary immune thrombocytopenia. Ann Hematol 100(9):2143–2154 10.1007/s00277-021-04590-0 PMC8310729 34308495

[7] Cheng CN, Yang YN, Yeh YH, Chen LW, Chen JS, Lin YC (2023) Predictors of remission in severe childhood immune thrombocytopenia. Diagnostics (Basel) 13(3):341 10.3390/diagnostics13030341 PMC9914323 36766447

[8] Li J, Tian J, Lu J, Wang Z, Ling J, Wu X, Yang F, Xia Y (2020) LncRNA GAS5 inhibits Th17 differentiation and alleviates immune thrombocytopenia via promoting the ubiquitination of STAT3. Int Immunopharmacol 80:106127 10.1016/j.intimp.2019.106127 31978798

[9] Naguib M, El Sawy S, Rashed L, AlHelf M, Abdelgwad M (2023) Long noncoding RNA maternally expressed gene 3, miR-125a-5p, CXCL13, and NF-kB in patients with immune thrombocytopenia. Genes Immun 24(2):108–115 10.1038/s41435-023-00200-3 PMC10110462 37045944

[10] El Zaiat RS, Abouelenin MAH, Saleh AA, El-Hawy M, Ahmedy IA, Mansour MM (2025) MiR-106b-5p, MiR-200c-3p, and MiR-146a-5p expression as putative biomarkers for disease state in primary immune thrombocytopenia. Expert Rev Hematol 1:8 10.1080/17474086.2025.2522298 40526093

[11] Jafarzadeh A, Marzban H, Nemati M, Jafarzadeh S, Mahjoubin-Tehran M, Hamblin MR, Mirzaei H, Mirzaei HR (2021) Dysregulated expression of miRNAs in immune thrombocytopenia. Epigenomics 13(16):1317–1327 10.2217/epi-2021-0092 34498489

[12] Tan JH, Ahmad Azahari AHS, Ali A, Ismail NAS (2023) Scoping review on epigenetic mechanisms in primary immune thrombocytopenia. Genes 14(3):555 10.3390/genes14030555 PMC10048672 36980827

[13] Li JQ, Tian JM, Fan XR, Wang ZY, Ling J, Wu XF, Yang FY, Xia YL (2020) MiR-106b-5p induces immune imbalance of Treg/Th17 in immune thrombocytopenic purpura through NR4A3/Foxp3 pathway. Cell Cycle 19(11):1265–1274 10.1080/15384101.2020.1746485 PMC7469554 32323598

[14] He Y, Ji D, Lu W, Li F, Huang X, Huang R, Chen G (2021) Bone marrow mesenchymal stem cell-derived exosomes induce the Th17/Treg imbalance in immune thrombocytopenia through miR-146a-5p/IRAK1 axis. Hum Cell 34(5):1360–1374 10.1007/s13577-021-00547-7 34052997

[15] Saraiva M, Vieira P, O’Garra A (2020) Biology and therapeutic potential of interleukin-10. J Exp Med 217(1):e20190418 10.1084/jem.20190418 PMC7037253 31611251

[16] Neumann C, Scheffold A, Rutz S (2019) Functions and regulation of T cell-derived interleukin-10. Semin Immunol 44:101344 10.1016/j.smim.2019.101344 31727465

[17] Toker H, Gorgun EP, Korkmaz EM, Yüce HB, Poyraz O (2018) The effects of IL-10 gene polymorphism on serum and gingival crevicular fluid levels of IL-6 and IL-10 in chronic periodontitis. J Appl Oral Sci 26:e20170232 10.1590/1678-7757-2017-0232 PMC5829549 29489938

[18] Kostić MS (2020) Immunomodulatory effects of TGF-β in the patients with immune thrombocytopenia. Acta Med Median 59(2):55–60

[19] Livak KJ, Schmittgen TD (2001) Analysis of relative gene expression data using real-time quantitative PCR and the 2(-Delta Delta C(T)) method. Methods 25(4):402–408 10.1006/meth.2001.1262 11846609

[20] Johnsen J (2012) Pathogenesis in immune thrombocytopenia: new insights. Hematology Am Soc Hematol Educ Program 1:306–312 10.1182/asheducation-2012.1.306 23233597

[21] Heitink-Pollé K, Nijsten J, Boonacker C, van der Sluis IM, van den Heuvel-Eibrink MM, de Haas M (2014) Clinical and laboratory predictors of chronic immune thrombocytopenia in children: A systematic review and meta-analysis. Blood 124:3295–3307 10.1182/blood-2014-04-570127 25305206

[22] Grimaldi-Bensouda L, Nordon C, Michel M, Viallard JF, Adoue D, Magy-Bertrand N, Durand JM, Quittet P, Fain O, Bonnotte B, Morin AS, Morel N, Costedoat-Chalumeau N, Pan-Petesch B, Khellaf M, Perlat A, Sacre K, Lefrere F, Abenhaim L, Godeau B (2016) Immune thrombocytopenia in adults: a prospective cohort study of clinical features and predictors of outcome. Haematologica 101:1039–1045 10.3324/haematol.2016.146373 PMC5060020 27229715

[23] Zakaria M, Beshir M, Hassan T, Esh A, Abdelaziz E, Tayib R, Nafea A (2023) Role of interleukin 4 (IL-4) and interleukin 6 (IL-6) in the pathogenesis and prognosis of childhood primary immune thrombocytopenia. Eur J Pediatr 182(7):3129–3138 10.1007/s00431-023-04945-x PMC10354134 37097445

[24] Lev P, Goette N, Marta R (2020) Pathophysiological mechanisms leading to low platelet count in immune thrombocytopenia. J Immunol Clin Sci 4(2):1–7

[25] Lassandro G, Palladino V, Palmieri VV, Amoruso A, Carlo De Vecchio G, Giordano P (2020) COVID-19 and children with immune thrombocytopenia: Emerging issues. Mediterranean Journal of Hematology and Infectious Diseases 12(1):e2020028 10.4084/MJHID.2020.028 PMC7202345 32395217

[26] Negash M, Tsegaye A, Medhin AG (2016) Diagnostic predictive value of platelet indices for discriminating hypoproductive versus immune thrombocytopenic purpura in patients attending a tertiary care teaching hospital in Addis Ababa. Ethiopia BMC Hematology 16(1):18 10.1186/s12878-016-0057-5 PMC4930598 27375850

[27] Arshad A, Mukry SN, Shamsi TS (2021) Clinical relevance of extended platelet indices in the diagnosis of immune thrombocytopenia. Acta Clin Croat 60(4):665–674 10.20471/acc.2021.60.04.14 PMC9196221 35734488

[28] Zuffere A, Kapur R, Semple JW (2017) Pathogenesis and therapeutic mechanisms in immune thrombocytopenia (ITP). J Clin Med 6(2):16

[29] Wang X, Li F, Li Y, Sun L, Meng Y, Fan X, Wang X, Wu D, Cheng Y, Hua F (2021) Decreased levels of immune-regulatory cytokines in patients with immune thrombocytopenia and long-lasting overexpression of these cytokines in the splenectomized patients. J Leukoc Biol 110(2):335–341 10.1002/JLB.5AB0521-621RR 34318945

[30] Metreveli S, Nanava N, Kvachadze I, Chikovani T, Janikashvili N (2022) TGF-β in patients with refractory immune thrombocytopenia. Georgian Scientists 4(5):1–8 32672688

[31] Wang L, Wang H, Zhu M, Ni X, Sun L, Wang W, Xie J, Li Y, Xu Y, Wang R, Han S, Zhang P, Peng J, Hou M, Hou Y (2024) Platelet-derived TGF-β1 induces functional reprogramming of myeloid-derived suppressor cells in immune thrombocytopenia. Blood 144(1):99–112 10.1182/blood.2023022738 38574321

[32] Goelz N, Bosch AMS, Rand ML, Eekels JJM, Franzoso FD, Schmugge M (2020) Increased levels of IL-10 and IL-1Ra counterbalance the proinflammatory cytokine pattern in acute pediatric immune thrombocytopenia. Cytokine 130:155078 10.1016/j.cyto.2020.155078 32220726

[33] Soliman MA, Helwa MA, Fath-Allah SK, El-Hawy MA, Badr HS, Barseem NF (2018) IL-10 polymorphisms and T-cell subsets could affect the clinical presentation and outcome of childhood immune thrombocytopenia in Egyptian population. APMIS 126(5):380–388 10.1111/apm.12823 29696721

[34] Liu XG et al (2015) Reduced CD4⁺CD25⁺Foxp3⁺ Treg/Th17 ratio in patients with primary immune thrombocytopenia. Clin Appl Thromb Hemost 21(6):513–521

[35] Liu L, Hua M, Liu C, He N, Li Z, Du D (2016) The aberrant expression of microRNAs and correlations with T cell subsets in patients with immune thrombocytopenia. Oncotarget 7:76453–76463 10.18632/oncotarget.12949 PMC5363522 27802180

[36] Qian C et al (2018) Differential expression of miR-106b-5p and miR-200c-3p in newly diagnosed versus chronic primary immune thrombocytopenia patients based on systematic analysis. Cell Physiol Biochem 45(1):301–318 10.1159/000486811 29402802

[37] Zhu Y, Zhu H, Xie X, Zheng Z, Ling Y (2019) MicroRNA expression profile in Treg cells in the course of primary immune thrombocytopenia. J Investig Med 67(8):1118–1124 10.1136/jim-2019-001020 PMC6900216 31273052

[38] Wang WF, Wang XS, Tan SY, Zhong LL, Chen J (2022) Expression of miR-106b—5p in children with primary immune thrombocytopenia and its correlation with T cells. Zhongguo Dang Dai Er Ke Za Zhi 24(4):411–416 10.7499/j.issn.1008-8830.2110139 PMC9044986 35527417

[39] Xie M et al (2020) MicroRNA-106b-5p inhibits regulatory T cell differentiation and promotes autoimmunity by targeting FOXP3. Autoimmun Rev 19(4):102491

[40] Nomair A, Mekky J, El-Hamshary S, Nomeir H (2023) Circulating miR-146a-5p and miR-132–3p as potential diagnostic biomarkers in epilepsy. Epilepsy Res 191:107089 10.1016/j.eplepsyres.2023.107089 36801489

